# Highlight: The Tree of Life Looking More Colorful

**DOI:** 10.1093/gbe/evu034

**Published:** 2014-03-04

**Authors:** Danielle Venton



Life is far more dynamic, varied, and exciting than we typically appreciate, genetically at least. Evolutionary biologists Angela Oliverio and Laura Katz believe epigenetics, polyploidy, and extensive genome reshuffling are found—not just in the strange corners of biology where exceptions are found—but across the whole tree of life, stemming as far back as the last universal common ancestor or LUCA.

In a new review in *Genome Biology Evolution*, Oliverio and Katz, both from Smith College, argue for a more expansive and inclusive view of biology and a turn away from what is typically taught in textbooks (Oliverio and Katz 2014).

“I like to study the weird, wonderful things in biology, things that didn’t read the textbook and don’t know how they’re supposed to structure their genome,” says Katz, whose lab at Smith studies eukaryotic genome evolution.

So widespread are the “nontextbook examples” of reproduction, ploidy number, and epigenetic interactions, in fact, that the authors suggest popular notions of LUCA may need an update. All of life uses DNA to produce RNA to produce proteins, a commonality known as the central dogma of biology. Because the same biochemical machinery is used in the path from DNA to proteins, it is reasonable to assume that LUCA used the same machinery. Also, because dynamic genome features can be found in each of the three main branches of life, perhaps they may have been part of LUCA’s genetic toolbox as well.

“The ability to do things like regulate the copied numbers of chromosomes and genome rearrangements while still inheriting full genome complements may have allowed LUCA to inhabit niches in new ways,” Katz says.

The authors examine three key ways in which genomes, bacterial ones especially, are unexpectedly dynamic: copied numbers of chromosomes, epigenetics, and variations in life cycle.

Though many bacterial species are usually thought of as having just one copy of a chromosome, they typically have many. (Generally this “polyploidy” isn’t like the polyploidy found in plants and animals, however, where distinct chromosomes are inherited from different parents.) For several bacteria, including *Escherichia coli*, the number of chromosomes varies with growth rate. When *Synechocystis* is growing exponentially, it has 218 genome copies. It still holds a considerable 58 copies in its linear growth phase. *Epulopiscium*, not to be outdone, copies its genome tens of thousands of times.

The advantage to all this extra copying may be to provide backups in the case of DNA damage or supporting a large cell size by allowing for gene expression to be regulated globally. It could also allow for certain copies to be inherited unchanged while other copies are recombined in novel experimentation.

In discussing epigenetics, the authors use an expansive definition coined by Denise Barlow, the woman who discovered the first imprinted gene in 1991: “Epigenetics has always been all the weird and wonderful things that can’t be explained by genetics.”

This includes regulated rearrangements in a genome, the incorporation of foreign material, and any heritable changes beyond changes in DNA sequence. For example, the eukaryotic ciliates Katz studies can make millions of chromosome copies. They take genes, scramble them into little pieces, and reform them into functional gene products. Showing a similar genetic juggling ability, some bacteria reshuffle genetic material to generate a diversity of antigens on the cell surface. These novelties help them escape host immune systems. The DNA of *Deinococcus radiodurans* can be ripped to shreds by radiation, and yet the organism is able to reassemble their genome as if it were no bother. Similar repair systems are found in archaea as well.

Among textbooks, bacterial and archaeal reproduction by binary fission is the common story, but one that is grossly oversimplified. Some bacteria reproduce by budding or multiple fissions, requiring coordinated inheritance of genetic material. Some bacterial and archaeal species can produce multiple internal offspring in a kind of “live birth.” Maternal DNA still operates in the “mother cell” and likely helps maintain metabolism while the daughter cells are growing. This life cycle calls for a clear replication and regulation of genome content and the capacity to mark and separate the genome to be inherited by the offspring.

Other bacteria reproduce by asymmetrical cell division, where one big cell spins off many smaller cells. If the genomes are not organized, marked, and separated out properly, daughter cells will be nonviable. Bacteria that reproduce by “budding” have the same ability to differentiate the “somatic” and “germline” genomes.

The paper may raise some eyebrows, Katz concedes. “The question is whether I’ve just selected the 25 freakish things in biology [that prove my point], or whether these 25 examples shed some light on properties that are widespread among organisms.”

However, evolutionary biologist Jonathan Eisen, a professor at the University of California, Davis, found nothing controversial in the paper.

“I completely agree that organisms across the tree of life have extremely dynamic, unusual, and varied genomes. Microbiologists have been talking about these ideas for many years, but I think for the most part people don't really appreciate this,” said Eisen.

He did note, however, that the authors use the terms “polyploidy” and “epigenetics” in a different way than biologists are accustomed to. Nonetheless he felt the review would be valuable for his teaching, as it gathers many examples of genomes behaving outside the perceived norm in one place.

“I hope this paper encourages us to examine our assumptions,”says Katz. “If we are open to other models, so that the textbook version of a genome dividing in a simple way is not the rule, but even the exception, then we might be able to better frame our questions and do better science.”
